# Threshold of Toxicological Concern—An Update for Non-Genotoxic Carcinogens

**DOI:** 10.3389/ftox.2021.688321

**Published:** 2021-06-24

**Authors:** Monika Batke, Fatemeh Moradi Afrapoli, Rupert Kellner, James F. Rathman, Chihae Yang, Mark T. D. Cronin, Sylvia E. Escher

**Affiliations:** ^1^Fraunhofer Institute for Toxicology and Experimental Medicine (ITEM), Hannover, Germany; ^2^Altamira, LLC, Columbus, OH, United States; ^3^Molecular Networks GmbH, Nuremberg, Germany; ^4^School of Pharmacy and Biomolecular Sciences, Liverpool John Moores University, Liverpool, United Kingdom

**Keywords:** DNA-reactive mutagenic, genotoxic, threshold, TTC, benchmark dose, risk assessment

## Abstract

The Threshold of Toxicological Concern (TTC) concept can be applied to organic compounds with the known chemical structure to derive a threshold for exposure, below which a toxic effect on human health by the compound is not expected. The TTC concept distinguishes between carcinogens that may act as genotoxic and non-genotoxic compounds. A positive prediction of a genotoxic mode of action, either by structural alerts or experimental data, leads to the application of the threshold value for genotoxic compounds. Non-genotoxic substances are assigned to the TTC value of their respective Cramer class, even though it is recognized that they could test positive in a rodent cancer bioassay. This study investigated the applicability of the Cramer classes specifically to provide adequate protection for non-genotoxic carcinogens. For this purpose, benchmark dose levels based on tumor incidence were compared with no observed effect levels (NOELs) derived from non-, pre- or neoplastic lesions. One key aspect was the categorization of compounds as non-genotoxic carcinogens. The recently finished CEFIC LRI project B18 classified the carcinogens of the Carcinogenicity Potency DataBase (CPDB) as either non-genotoxic or genotoxic compounds based on experimental or *in silico* data. A detailed consistency check resulted in a dataset of 137 non-genotoxic organic compounds. For these 137 compounds, NOEL values were derived from high quality animal studies with oral exposure and chronic duration using well-known repositories, such as RepDose, ToxRef, and COSMOS DB. Further, an effective tumor dose (ETD10) was calculated and compared with the lower confidence limit on benchmark dose levels (BMDL10) derived by model averaging. Comparative analysis of NOEL/EDT10/BMDL10 values showed that potentially bioaccumulative compounds in humans, as well as steroids, which both belong to the exclusion categories, occur predominantly in the region of the fifth percentiles of the distributions. Excluding these 25 compounds resulted in significantly higher but comparable fifth percentile chronic NOEL and BMDL10 values, while the fifth percentile EDT10 value was slightly higher but not statistically significant. The comparison of the obtained distributions of NOELs with the existing Cramer classes and their derived TTC values supports the application of Cramer class thresholds to all non-genotoxic compounds, such as non-genotoxic carcinogens.

## Introduction

In 2004, Kroes et al. derived TTC values for potentially genotoxic substances from Carcinogenicity Potency DataBase (CPDB) (Cheeseman et al., 1999; Gold et al., 1999). In the resultant decision, three potentially genotoxic compounds were defined as DNA-reactive mutagens. DNA-reactive mutagens are genotoxic substances that can modify DNA directly, either as the parent chemical or in the form of a metabolite (Boobis et al., 2017). They have the potential to directly cause DNA damage when present at low levels, leading to mutations and, therefore, potentially causing cancer. This type of mutagenic carcinogen is usually detected in Organisation for Economic Cooperation and Development (OECD), Guidelines for the Testing of Chemicals, such as the bacterial reverse mutation (mutagenicity) assay. Further data that contribute to the classification are mouse lymphoma assay or *in vivo* micronucleus test. In case of missing data, the evaluation can be supported by rule-based and statistical quantitative structure–activity relationship (QSAR) models. The current TTC value for compounds with a structural alert for DNA reactivity or experimental data for genotoxicity is 0.025 μg/kg/d. Since the TTC limit for DNA-reactive genotoxic substances is far below all other TTC values, the identification of experimental data or structural alerts for genotoxicity is one of the first decision points of the current TTC decision tree (EFSA WHO, 2016; More et al., 2019).

In the case of non-directly DNA-reactive genotoxic substances, a mechanism of action is assumed where a threshold for tumor formation can be established. The most sensitive effect(s) after chronic exposure of non-DNA reactive compounds might, however, either be non-neoplastic or neoplastic effects, depending on their mode of action. Within the TTC concept, these substances, like all other chronically applied toxins, are evaluated using the Cramer classification tree and the application of the respective TTC values. This approach is supported by European Food Safety Authority (EFSA) opinions of 2012 and 2016 (EFSA, 2012; EFSA WHO, 2016). In 2012, EFSA compared (EFSA, 2012) the TD50 values of genotoxic and non-genotoxic carcinogens from the Carcinogenicity Potency DataBase (CPDB) and confirmed the finding of Cheeseman et al. (1999) that non-genotoxic compounds are less potent than genotoxic compounds.

As outlined by Boobis et al. (2017), the TTC value for genotoxic carcinogens is currently based on a linear extrapolation to a virtual safe dose, using one in one million lifetime tumor risk levels in exposed individuals as target risk. Boobis et al. (2017) pointed out that the major difference between the derivation of the TTC for genotoxic substances and those for the Cramer classes is the level of protection [which equals dividing the point of departure (POD) by 10^6^] for values of genotoxic carcinogens vs. 10^2^ for the chronic PODs of the Cramer classes. Reducing the safety factor of the TTC values for genotoxic substances by three orders of magnitude (i.e., using a margin of 10^3^) would result in a TTC value of 2.5 μg/kg/d, which is in the same range as the Cramer class 3 value of 1.5 μg/kg/d. Despite this estimation, Boobis et al. (2017) proposed the derivation of TTC values for genotoxic (DNA reactive) and non-genotoxic compounds following analysis of freshly curated carcinogen datasets.

The current analysis focused on non-genotoxic carcinogens, since none of the previous analyses provided proof that the application of Cramer class thresholds is safe for them. TTC values for non-genotoxic carcinogens are currently derived using the existing thresholds for the different Cramer classes. To achieve the goal of this analysis, different points of departure for non-genotoxic carcinogens were compared to identify the most sensitive, and thus, most appropriate values for threshold derivation.

In the LRI B18 2 project, the *in vivo* dataset for TTC was extended with data from high quality (sub)chronic *in vivo* studies for substances determined to be non-genotoxic carcinogens in a rodent bioassay. The most sensitive dose descriptors from these (sub)chronic studies were compared with benchmark doses derived from tumor incidences; thereafter, they were compared with the NOEL values of the actual Cramer classes in terms of distributions and possible TTC values. Finally, the use of Cramer class thresholds for non-genotoxic carcinogens is discussed.

## Materials and Methods

### Derivation of a Dataset of Non-genotoxic Carcinogens

The dataset comprised 137 carcinogens derived originally from the CPDB database (Cheeseman et al., 1999). These compounds are identified as non-genotoxic carcinogens based on a decision tree (CEFIC LRI B18 project) and a consistency check taking into account further peer-reviewed publications. Publications with any kind of tumor being significantly increased over the background because of chemical exposure were considered. All kinds of rodent bioassays were considered; and in case of conflicting results, decisions were based on a weight of evidence approach considering further mechanistic information. Literature on genotoxicity was searched for in PubChem^1^ and Pubmed^2^ by substance name and the terms “genotoxicity,” “carcinogenicity,” or “mutagenicity.” Furthermore, european chemicals agency (ECHAChem), national toxicology progamm (NTP), and international agency for research on cancer (IARC) reports were considered. Substances with a mode of action irrelevant to humans were excluded. Most prominent examples of substances excluded were such substances inducing forestomach tumors in rodents (Proctor et al., 2007) or thyroid stimulating hormone (TSH)-induced thyroid tumors (Bartsch et al., 2018). Additionally, we added information on IARC (2021) classification for all substances if available.

The CPDB documents tumor incidences per observed tumor type, organ, gender, and dose group compared with study controls and, if available, historical controls. The tumor incidences were used to calculate the BMDL10 and ETD10 values and include studies on different species, such as dog, hamster, and monkey. The CPDB comprises 513 studies for the 137 chemicals. However, the cancer bioassay study design is often focused on tumor detection alone, so non-neoplastic effects are insufficiently investigated. To enable an analysis of NOEL values, OECD guideline-conformant (sub)chronic toxicity studies were collected from high quality databases [RepDose (https://repdose.item.fraunhofer.de), ToxREF DB (Martin et al., 2009), Cosmos (Yang et al., 2017)] or from peer-reviewed publications. The purpose of this analysis was to extend the study reports of the CPDB with observed non-neoplastic effects, in particular the lowest observed effect levels (LOELs) per gender, organ, and effect. This search aimed to integrate at least one chronic study per chemical in the dataset, especially for chemicals for which only cancer studies were available in the CPDB.

A study quality measure assessing the study reliability in terms of guideline alignment (as recorded in RepDose) was assigned to each study to distinguish guideline-compliant studies from studies of lower quality. In this context, the studies of lower quality included cancer studies that miss other than cancer-related chronic endpoints, studies with major deviations from guidelines, as well as studies with a special focus and, thus, deviating from the scope of the examination.

The final dataset of 137 chemicals comprised 525 studies with repeated exposure: 190 subchronic (83–99 days) and 259 chronic toxicity studies, and 76 cancer studies (>350 days). Rats or mice were mainly tested orally, or, in a very few cases, by inhalation (*N* < 5). In the case of inhalation, the route was selected because of the volatility of the substances and 100% absorption of the nominal concentration was assumed. The concentrations administered in the inhalation studies were included as body dose converted to mg/kg bw/d.

### The Points of Departure

#### No Observed Effect Level Values

The respective NOEL values were calculated by the use of an extrapolation factor of 3 for LOEL to NOEL, to minimize the impact of differences in study design like dose spacing (EFSA, 2012). The NOEL values were collected without further consideration of adversity of effects from the RepDose database.

- The LOEL values were derived from (sub)chronic studies, considering three different types of significantly increased effects in the study:
- the lowest observed effect level of the overall study (LOEL)
- the lowest observed effect level of the most sensitive tumor (neoplastic LOEL) and
- the lowest observed effect level of the most sensitive non-neoplastic effect, defined as effect not diagnosed as a tumor (non-neoplastic LOEL)

Allometric scaling was applied to account for interspecies differences with a factor of 4 for rats and 7 for mice (EFSA, 2012). An extrapolation factor of 2 was applied for subchronic to chronic extrapolation (ECHA, 2012) to compare the outcome of different study durations. Out of these standardized NOEL values, the lowest value per substance is considered. The preferred unit of the standardized NOEL values is mmol/kg bw/d; and for final comparison with published datasets, values are, in addition, given in mg/kg bw/d.

The first analysis of NOELs assessed the influences of study quality and study duration on the respective fifth percentile of the distribution of the NOELs. In the next step, we compared the fifth percentile for the overall NOELs, and those for non-neoplastic lesions and neoplastic tumors. The NOEL distributions deriving the lowest fifth percentile were used for the following steps. The resulting NOEL values per compound are provided in Supplementary Table 1.

#### Effective Tumor Dose 10 Values

The effective tumorigenic dose for 10% of the animals for the most sensitive tumor was determined by the previous project LRI B18 (publication in preparation) on the same chemicals but a different study repository was used. The inclusion criteria for studies and their respective tumors were oral route, two or more dose levels in addition to the control group, specific tumor site, and quality-controlled evidence for human relevant carcinogenicity. A significant relationship between dose and tumor counts was analyzed based on contingency tables. The doses of these tumors were fitted using logistic regression to the proportion of tumor-bearing animals with a logit link function. The Akaike information criterion (AIC) was used to characterize the goodness of fit of the logistic regression model to dose-response of the data set (AICnull). Studies with values of AICnull—AIClogistic > 2 were used for further analyses. To allow comparison with other PODs, allometric scaling factors were applied to the ETD10 values, which were 4 (rat); 7 (mouse), 1.4 (dog), 5 (hamster), and 2 (monkey) (EFSA, 2012).

The resulting ETD10 values per compound are provided in Supplementary Table 2.

#### Benchmark Dose Level 10 Values

The same study inclusion criteria outlined above for the modeling of ETD10 were applied to calculate BMDL10. A benchmark response (BMR) of 10% was predefined as the critical effect size. BMDL10 values were standardized for allometric differences as described above.

Model averaging was used to estimate the dose (BMDL_10_) associated with the specified effect [within the PROAST software (version 64.10; (Slob, 2002)]. The average BMD_10_ was estimated from the complete dose–response dataset by fitting to the available nine different dose–response models, which are named according to their fitting functions as two.stage, log.logist, weibull, log.prob, gamma, probit, logistic, expon, and hill. Statistical uncertainties in the data are taken into account in the 90th confidence interval around the BMD, the lower limit of which (denoted as BMDL10) is the POD for risk assessment (Hardy et al., 2017). For further reference, please refer to https://www.rivm.nl/en/proast.

The resulting BMDL values per compound are provided in Supplementary Table 2.

### Robustness of Threshold of Toxicological Concern Values

#### Derivation of Threshold of Toxicological Concern Values

Before calculating the TTC value based on the fifth percentile of the POD distributions, we investigated by manual inspection of all chemicals up to the 30th percentile whether specific chemical classes are associated with low NOEL values and, therefore, influence the fifth percentile. We analyzed the impact of these chemical classes on the fifth percentile in comparison to leaving out the same number of randomly selected chemicals. A KNIME workflow was built to calculate the fifth percentile [type 8 (Hyndman and Fan, 1996)] leaving out a randomly predefined percentage of substances with 100 repetitions. A random leave out with 5/10/20 iterations excludes randomly 20/10/5% of the substances from the whole dataset and calculates the respective fifth percentile of the remaining 80/90/95% of the compounds. After 5/10/20 iterations, each compound was excluded once. The lowest and highest fifth percentiles calculated within the 100 iterations are recorded as possible range after random leave out. In the case that the fifth percentile, after exclusion of a specific group of chemicals, exceeds the range of TTC values that a random set is left out, there is high probability that this substance group has a specific impact on the remaining data set.

In order to derive TTC values, we assigned all the 137 substances to their respective Cramer classes using QSAR Toolbox 4.3 and Toxtree v3.1.0.1851. There are deviations between the two implementations and the original and extended Cramer rules for 27 substances (19% overall, explicit data not shown). The ranges of discrepancies are similar to those observed by Bhatia et al. (2015). As discrepancies of this proportion are not acceptable, we compared the concordance of the originally published classification by Munro according to Cramer with the classifications assigned by Toxtree and QSAR Toolbox: Toxtree resulted in 66/607 (11%) original Munro DB chemicals with deviating classifications compared with those assigned originally, whereas QSAR Toolbox classifications deviated in 40/607 (7%) chemicals only. With both tools, the so-called “original Cramer classification” was used. Based on these results, the original Cramer classification by the QSAR Toolbox was used in this project.

In the final step to derive a TTC value, we extrapolated the fifth percentile to human exposure thresholds. As species differences were accounted for by allometric scaling prior to the derivation of fifth percentiles, the remaining extrapolation accounted for the remaining uncertainty with respect to interspecies differences (a factor of 2.5) and the intraspecies variability (factor of 10) (Escher et al., 2010). This approach differs from that proposed by Munro et al. (1996). They applied a default safety factor of 100 to the fifth percentiles based on a margin of safety concept. NOEL values from studies with less than chronic exposure were additionally adjusted by a factor of 3. The consideration of the time difference in NOEL values was not necessary in the current analysis, which is based on chronic studies only.

#### Comparison of Distributions of Different Points of Departure

The distributions of the log-normal values for NOELs, BMDLs, and ETD10s were plotted in parallel histograms. This qualitative comparison of the distributions is accomplished by derivation of the respective TTC values and their confidence interval.

#### Statistical Analyses and Special Diagrams

Statistical analyses were performed using R [version 3.5.1 (2018-07-02)]. The fifth percentiles are calculated using the quantile type 8 function (Hyndman and Fan, 1996). Additional libraries ggplot2 and ggridges were used for the respective plots. KNIME was used for the leave-out workflow only.

## Results

### Dataset and No Observed Effect Level Values

The TTC dataset comprised 137 non-genotoxic compounds. All the 137 compounds had at least one chronic toxicity study. The majority (98 substances, 72%) has, at minimum, one high quality chronic study, whereas 39 substances are described by lower quality chronic toxicity studies alone. In addition, cancer studies are available. The cancer studies were rated as being of lower quality for the derivation of NOEL values, as they are designed to detect carcinogenic effects and, thus, most often apply relatively high doses without reporting on the non-neoplastic effects, sometimes even focusing their results on the major tumor-target organs.

#### Influence of Study Quality and Duration on the NOEL

To assess the impact of study quality and study duration on the fifth percentile, data sets containing the same chemicals were compared. This approach eliminates the potential influence of different chemical domains. Fifth percentiles are similar for high (5 × 10^−5^) and lower (1 × 10^−5^) quality studies as indicated by the overlapping confidence intervals (CI 95%). Also, the study duration has no significant impact on the fifth percentiles as subchronic (4 × 10^−5^) and chronic (1 × 10^−5^) fifth percentiles showed widely overlapping CIs (data not shown).

#### Neoplastic and Non-neoplastic Effects Determine the NOEL

**Figure 1:**
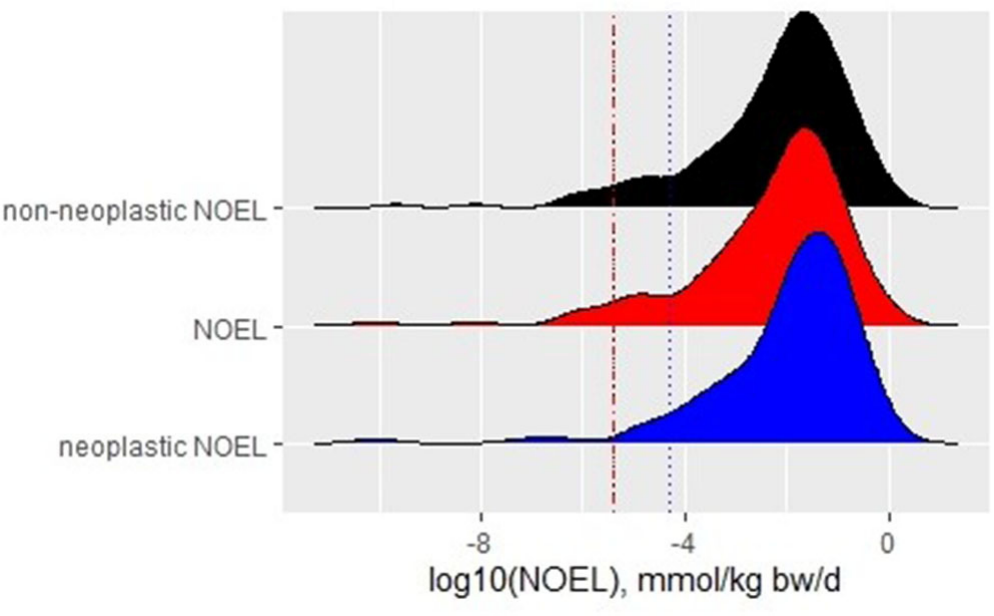
Density plot with histograms for NOELs derived for different endpoints with fifth percentile of each distribution represented by a dotted line of the respective color.

A slight shift to higher values is observed for the neoplastic NOELs compared with non-neoplastic and overall NOEL values (Figure 1). This finding raises the question of whether the data support the widely spread thesis that in rodent bioassay tumors occur at higher doses, or even only at the highest tested dose (HTD) (Anisimov et al., 2005; van der Laan et al., 2016). In the dataset, tumors occurred in 55% of the compounds at the LOEL of the study. Tumor effects in isolation determined the LOEL in 11% of all substances (whereas 44% showed both non-neoplastic and neoplastic effects at the LOEL). Thirty-three percent of the substances showed tumors only at the HTD but other effects at lower doses. NOELs for the overall study as well as non-neoplastic NOELs show the fifth percentile of 4 × 10^−6^ mmol/kg/d, whereas the fifth percentile of neoplastic NOELs is slightly higher with 3 × 10^−5^ mmol/kg/d. The 95% CIs are identical for the non-neoplastic NOELs and the NOELs. For neoplastic NOELs, the 95% CI still overlaps with those of the other two but is one order of magnitude broader, reaching to higher values (see Figure 1, data for 95% CIs are not shown).

This analysis supports the observation of a broad overlap of all NOEL distributions but about half of the substances included in the analysis have neoplastic NOELs that are higher than the overall NOEL. The overall NOELs of the chronic studies are, thus, a conservative estimate for a POD.

### Robustness of Threshold of Toxicological Concern Values

#### Derivation of a Threshold of Toxicological Concern Value

**Figure 2:**
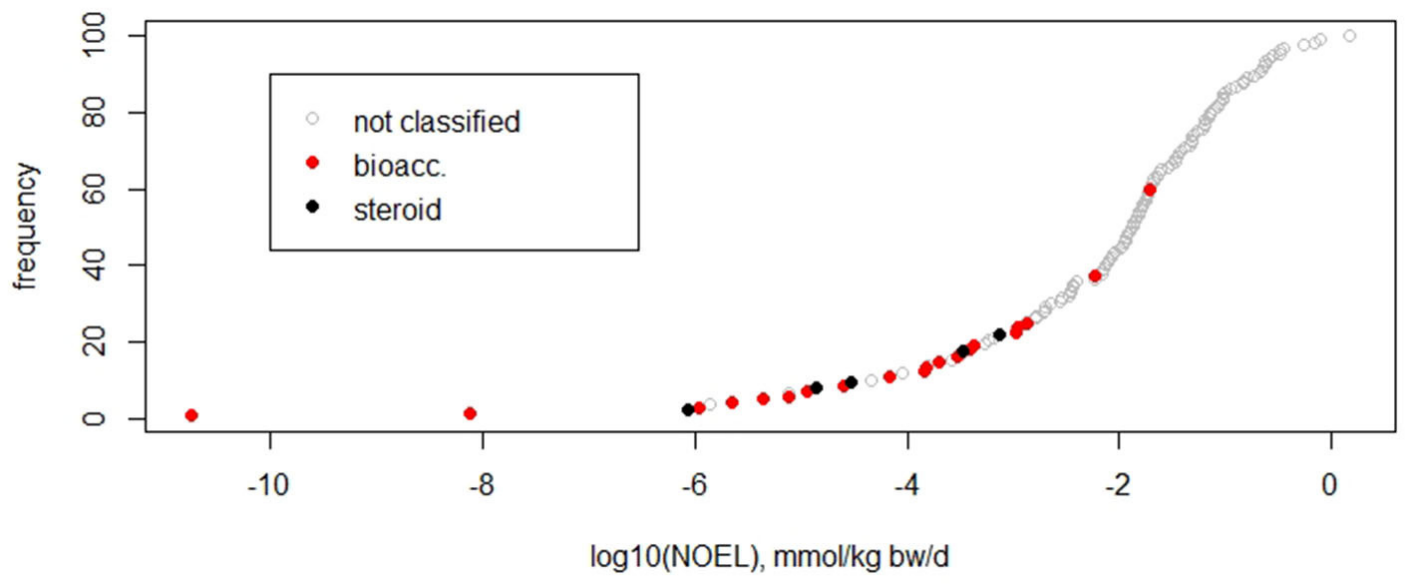
Cumulative frequency plot of NOEL values indicating subgroups of compounds, such as bioaccumulating in humans (bioacc) and steroids.

As the dataset serves to test whether or not the current TTC concept is applicable to non-genotoxic carcinogens, it should not include compound classes that are *a priori* excluded from the applicability domain of the TTC concept (Kroes et al., 2004; EFSA WHO, 2016). Within the dataset of 137 substances, 20 compounds show bioaccumulating properties in humans (identified by experimentally measured half lives *in vivo* as described by Corie Ellison et al. in preparation), and five compounds are steroids (identification by steroid-like structure and activity). These two classes are outside of the applicability of the TTC concept, and, additionally, the steroids belong to the cohort of concern. Bioaccumulating substances may, however, exhibit excess toxicity because of their long-term stability and result in high cumulative doses in humans, whereas steroids are suspected to show adverse effects at very low doses. The influence of these compounds on the fifth percentile was analyzed by excluding them and comparing the resulting fifth percentiles to randomly removing the same number of substances. Exclusion of the bioaccumulative substances increased the fifth percentile by a factor of 10, from 5 × 10^−6^ to 5 × 10^−5^ mmol/kg/d. This increase is higher than when randomly removing a similar number of compounds (20%), which results in a range of 2 × 10^−6^ to 2 × 10^−5^ mmol/kg/d (Table 1). Subsequent exclusion of five steroids resulted in the fifth percentile of 3 × 10^−4^ mmol/kg bw/d, which is again higher than the range of 2 × 10^−6^ to 2 × 10^−5^ mmol/kg bw/d achieved by randomly removing the same number of compounds. The cumulative frequency diagram of NOELs shows that bioaccumulators and steroids are mainly found at the lower end (Figure 2).

**Table 1:** Impact of two different compound classes on the fifth percentiles in the NOEL dataset; ID 1- all NOEL values (*N* = 137 cmpds); exclusion of bioaccumulating compounds (ID 2; *N* = 117; reduction by 20%) and subsequently steroid-like compounds (*N* = 112; additional reduction by 5%).

**ID**	**Dataset**	***N***	**5th percentile (mmol/kg /d)**	**% randomly left out**	**5th percentile after random removal**
1	NOEL	137	5 × 10^−6^	n.a.	n.a.
2	-bioacc.	117	5 × 10^−5^	20	2 × 10^−6^−2 × 10^−5^
3	-bioacc, -steroids	112	3 × 10^−4^	5	4 × 10^−5^-2 × 10^−4^

*The fifth percentiles after exclusion of bioaccumulating and steroid compounds are above those from removing a comparable number of randomly selected compounds, which indicates that these substance classes have very low NOELs and are highly toxic*.

This analysis suggests that potentially bioaccumulating compounds and steroids are predominantly found at the lower end of the NOEL distribution, pushing the dataset toward lower fifth percentiles. These classes should be excluded from the datasets in order to derive relevant thresholds for compounds in the applicability domain of the approach.

A similar analysis was carried out with the BMDL values obtained by model averaging (Table 2, BMDL_10_). The analysis discriminates BMDL_10_ values derived from studies with two or more dose groups in addition to control (ID 1) from those based on studies with three or more dose groups in addition to control (ID 2). The results are in agreement with the results obtained for NOELs (Table 1).

**Table 2:** Overview on the fifth percentiles obtained from the BMDL_10_ datasets: ID 1 BMDL_10_ (*N* = 116 cmpds); ID1.1 -bioacc, -steroids (*N* = 100; reduction by ~15%); ID2 BMDL_10_ based on studies with at least three dose groups tested (*N* = 62); ID2.1 -bioacc, -steroids (*N* = 51; reduction by ~20%).

**ID**	**Dataset**	***N***	**5th percentile (mg/kg/d)**	**5^**th**^ percentile after random removal**
1	BMDL_10_	116	6 × 10^−3^	5 × 10^−3^ – 2 × 10^−2^ (15%)
1.1	-bioacc, -steroids	100	4 × 10^−2^	n.a.
2	BMDL_10_^*^	62	4 × 10^−3^	4 × 10^−3^ – 3 × 10^−2^ (20%)
2.1	-bioacc, -steroids^*^	51	3 × 10^−2^	n.a.

*The fifth percentiles after exclusion of bioaccumulating and steroid compounds exceed those after removing a comparable amount of randomly selected compounds (15 and 20%, respectively), which indicates that these substance classes have very low NOELs and are highly toxic. The fifth percentiles of ID1.1 and 2.1 show comparable results*.

* *Studies with more than three dose groups tested*.

#### Comparison of the Fifth Percentiles for No Observed Effect Level, Effective Tumor Dose 10, and Benchmark Dose Level 10 Values

**Figure 3:**
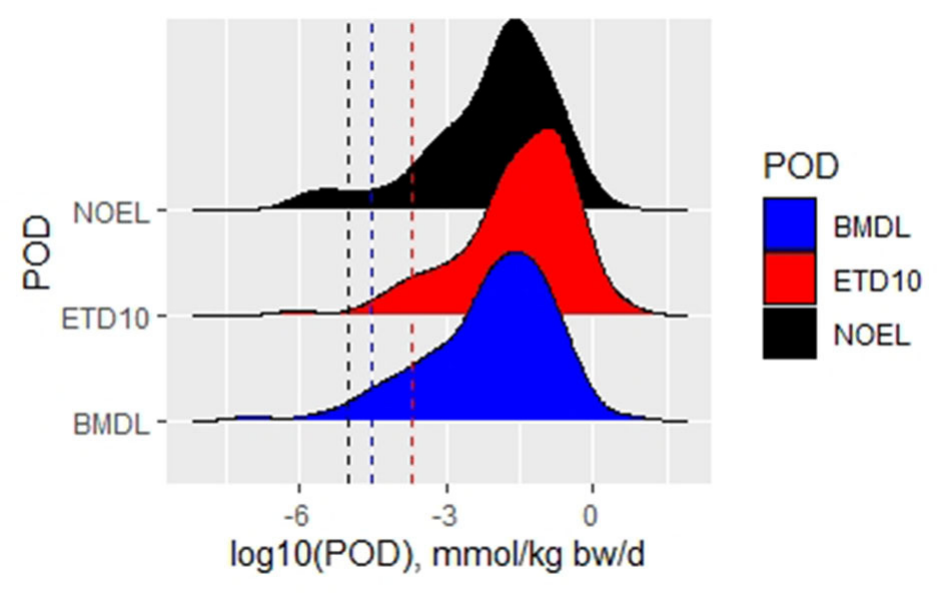
Density plot with histograms for different types of PODs with indication of the respective fifth percentiles by dotted lines in respective colors.

A comparative analysis of overall NOELs, ETD_10_, and BMDL_10_ values was performed to identify the most sensitive dose descriptors for non-genotoxic compounds (Figure 3, Table 3). ETD10 values were available for 118 substances and led to the fifth percentile of 6 × 10^−2^ mg/kg/d. BMDL_10_ values for tumor types being tested in studies with two or more dose levels were calculated for 116 compounds. A BMDL_10_ value based on studies with three or more dose levels was available for 62 substances. Taking into account that benchmark dose modeling is dependent on a dose–response curve, the dataset based on studies with three or more dose groups (plus controls) is generally considered more reliable than studies testing two dose groups. Nevertheless, both BMDL10 datasets derived similar fifth percentiles, with 4 × 10^−2^ and 3 × 10^−2^ mg/kg/d after exclusion of bioaccumulators and steroids (Table 2).

**Table 3:** Fifth percentiles for NOEL, ETD10, and BMDL10 values.

**All available values**	***N***	**5th percentile (mg/kg/d)**	**5th percentile (mmol/kg /d)**
**Dataset**			
NOEL	137	1.9 × 10^−3^	5 × 10^−6^
ETD10	118	6.0 × 10^−2^	1 × 10^−4^.
BMDL10	116	6.5 × 10^−3^	3 × 10^−5^.
**Same Chemicals**			
NOEL	101	5.6 × 10^−3^	9 × 10^−6^
ETD10	101	7.1 × 10^−2^	2 × 10^−4^
BMDL10	101	7.3 × 10^−3^	3 × 10^−5^

These datasets for ETD10 and BMDL10 do not contain exactly the same compounds, and this difference might interfere with a direct comparison of PODs. Therefore, we compiled a dataset of 101 chemicals for which all three types of POD were available (Figure 2, Table 3). The comparative analysis shows that for the same substances, the fifth percentile is most conservative using the overall NOEL values or the BMDL_10_. The fifth percentile in the EDT_10_ dataset is slightly but not significantly higher.

The NOEL values are the largest dataset comprising all the 137 non-genotoxic compounds. Since the comparison of PODs revealed NOELs to be a conservative estimate, the fifth percentile NOEL value is used in the following to derive TTC values.

#### Cramer Classifications and Threshold of Toxicological Concern Values

**Figure 4:**
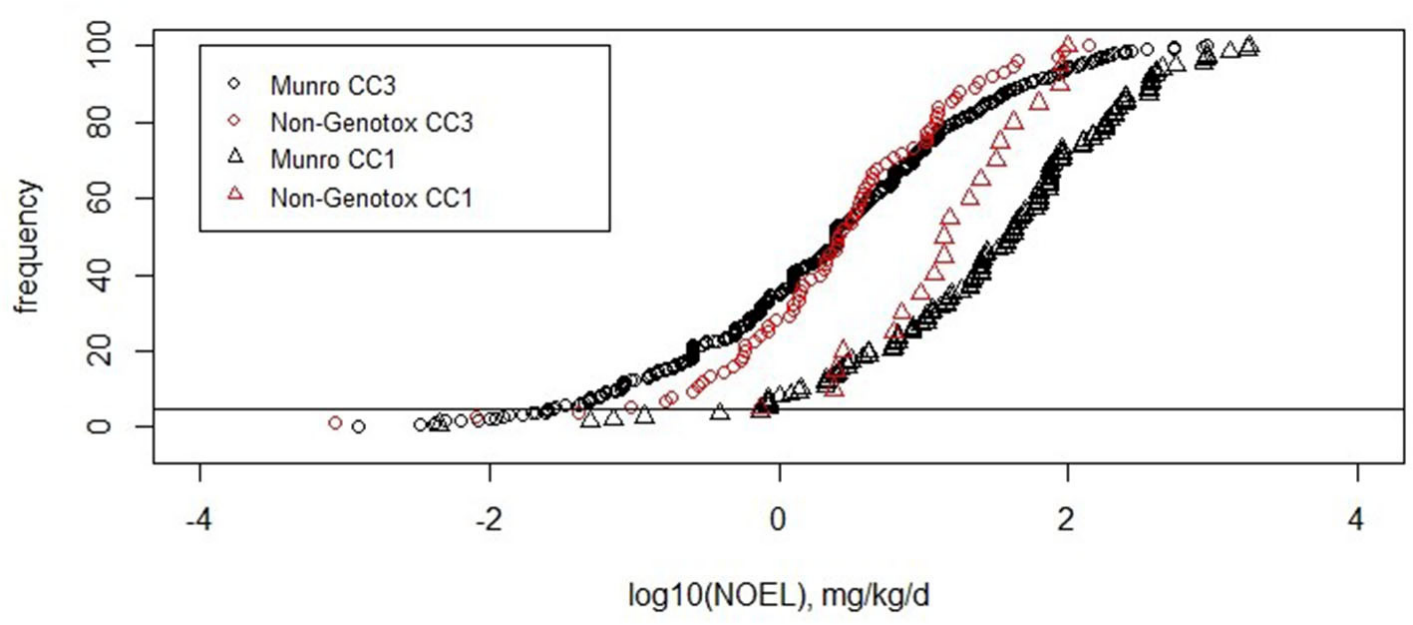
Cumulative frequencies of the original Munro data and non-genotoxic dataset for Cramer classes 1 and 3.

Most of the non-genotoxic carcinogens belong to Cramer class 3. Out of the 137 substances, 114 belong to Cramer class 3, 5 to Cramer class 2, and 18 to Cramer class 1. The exclusion of the potentially bioaccumulating substances and the steroids mostly affects Cramer class 3, which is reduced to 90 compounds. These 90 compounds show the fifth percentile of 5 × 10^−2^ mg/kg/d (Table 4). The distribution of the values is similar to the original Munro data for Cramer classes 3 and 1 as shown in Figure 4.

**Table 4:** Fifth percentiles of non-genotoxic datasets and original datasets by Munro for Cramer class 3 with respective ranges after randomly removing 5%.

**Dataset**		**mg/kg bw/d**		**mmol/kg bw/d**	
**NOEL**	***N***	**5th P**	**Range**	**5th P**	**Range**
-bioacc, -steroids	112	6 × 10^−2^	5 × 10^−2^- 2 × 10^−1^	3 × 10^−4^	4 × 10^−5^ – 2 × 10^−4^
Class 3	90	5 × 10^−2^	5 × 10^−2^- 1.5 × 10^−1^	2.5 × 10^−4^	2 × 10^−4^ – 4 × 10^−4^

For Cramer class 3, the number of substances in the non-genotoxic dataset is sufficient to derive a TTC value, which turns out to be similar to the original Cramer class 3: 2 μg/kg/d for non-genotoxic compounds (range of 2–6 μg/kg/d) and 1 μg/kg/d (range of 2–7 μg/kg/d) for the original Munro data. The ranges here are a measure of robustness toward random removal of 5% of the substances (Table 5).

**Table 5:** TTC values of non-genotoxic datasets and original datasets by Munro for Cramer Class 3 with respective ranges after randomly removing 5%.

**TTC values**	***N***	TTC^*^ (μg/kg/d)	**Range**	TTC^*^ (μmol/kg/d)	**Range**
Class 3	90	2	2, 6	1 × 10^−2^	9 × 10^−3^ – 2 × 10^−1^
Munro Class 3	448	1	1, 1.3	2 × 10^−2^	9 × 10^−3^ – 2 × 10^−2^

* *Calculated fifth percentile/25 as allometric scaling was applied to the NOEL before comparison*.

## Discussion

Like all other substances remaining after exclusion of genotoxicants and those generally excluded from TTC, non-genotoxic compounds are currently assigned to the Cramer classes. This approach is based on the observation that tumor formation is one of several chronic adverse effects and that the underlying mechanisms show a threshold (Clewell et al., 2019). This approach points to the question of whether non-genotoxic carcinogens, in the context of TTC, are best described by their most sensitive tumor or by their non-neoplastic adverse effects and which dose descriptor is the most conservative.

The dataset used in this study was subject to a consistency check, which included the exclusion of non-carcinogenic compounds (Cefic, 2018) and non-human relevant tumor types as well as an allocation of the mode of action (non-genotoxic vs. genotoxic) by extensive literature review on available experimental and predicted data.

Different points of departure were compared based on either tumor incidences (EDT_10_, BMDL_10_) or the NOELs. The TD50 values used in the original CPDB database are not considered to be conservative measures as they are based on a 50% tumor rate. Furthermore, linear extrapolation to a virtual safe dose with a tumor risk of 1–1,000,000 implicates high uncertainty. Thus, the ETD10 values replace the TD50 values in this analysis.

This approach showed that the fifth percentiles derived from the BMDL_10_, EDT_10_, and NOEL value distributions across > 100 carcinogens do not differ significantly indicating that for this set of compounds tumor formation does not, *per se*, occur at lower doses than those for non-neoplastic lesions. This is in line with findings from a previous study, which showed that BMD confidence intervals for tumor responses correlate with NOEL values from (sub)chronic toxicity studies (Braakhuis et al., 2018). Any of the three PODs could, thus, have been used to calculate the TTC value for non-genotoxic compounds. In actuality, the NOELs were used, since these values were available for more compounds than the BMDL10 and EDT10 values.

The comparison of NOEL values for tumors and non-neoplastic effects, as depicted in Figure 1, supports the conclusion of the NOEL as the most conservative value. For most of the substances (89%), LOELs are determined from non-neoplastic effects, with half of the substances showing tumors at this dose level. These LOELs are below the HTD (maximal tolerated dose) for 67% of all the chemicals in this dataset. Since several authors (Gaylor, 2005; Goodman, 2018) have argued that the classical two-year bioassay by NTP will result in tumors at the HTD, it is interesting to note that within this dataset tumors do not primarily start to occur at the highest dose tested. The observation of predominant non-neoplastic lesions supports the hypothesis for the mode of actions of non-genotoxic compounds made by Braakhuis et al. (2018). They stated that exposure to low doses of non-genotoxic carcinogens will change some biological processes slightly, whereas the repeated exposure will lead to overt disturbances, e.g., in hormonal balance or redox equivalents, inducing neoplastic changes, such as increased cell proliferation (Braakhuis et al., 2018).

NOEL values of subchronic studies were additionally compared with those of chronic studies to compensate for possible deficiencies in the long-term study. Within this comparison, the differences between the NOELs were not higher than the well-established extrapolation factor of 2 for subchronic to chronic exposure (EFSA, 2012). The data support the hypothesis that there is no significant added value of chronic studies neither with respect to POD (Braakhuis et al., 2018) nor concerning the predictability of the effect (van der Laan et al., 2016; Woutersen et al., 2016) for non-genotoxic carcinogens. Nevertheless, we used the chronic dataset for threshold derivation simply, as it was larger than that for subchronic effects and covers a greater number of compounds.

It has been hypothesized that there may be a minimal (e.g., 5–10%) response at the NOEL as opposed to the complete absence of an effect. This discrepancy of the effect is most likely due to the limited statistical power of the animal studies (Hardy et al., 2017; Braakhuis et al., 2018). As shown in Figure 3, the data set has no notable differences between the NOEL and the BMDL10, supporting this hypothesis. Both values will feed into the final analysis to derive TTC values.

### Derivation and Assessment of Threshold of Toxicological Concern Values for Cramer Classes

The assignment of Cramer classes to the dataset is a prerequisite for the derivation of the respective TTC values for Cramer classes 3 and 1. The shortcomings and ongoing improvements of Cramer classifications using the OECD Toolbox or Toxtree have already been well-documented in previous publications (Bhatia et al., 2015; Roberts et al., 2015; Boobis et al., 2017; Yang et al., 2017). As we aimed to compare the TTC values to the original values derived by Munro, we decided to use the original Cramer decision tree from the OECD Toolbox for the classification of the data set.

Most of the compounds in the dataset were assigned to Cramer class 3. As commonly occurs, very few or no compounds were assigned to Cramer class 3. As commonly occurs, very few or no compounds were assigned to Cramer class 2 and for this dataset only 18 compounds into Cramer class 1 (Patel et al., 2020). The determination of a TTC value for Cramer class 1 compounds was, thus, not reasonable, as the fifth percentile would be based on a single substance only. The NOEL values of the 18 non-genotoxic carcinogens are consistently distributed within the Cramer class 1 dataset (Figure 4). This preliminary analysis is an indication that the Cramer class 1 threshold can be applied to non-genotoxic compounds. The TTC of Cramer class 3 on non-genotoxic carcinogens is 2 μg/kg/d after exclusion of the possibly bioaccumulating substances and steroids. The original Munro data derive a very similar TTC value of 1 μg/kg/d. Other datasets, such as the COSMOS DB, for cosmetics show a TTC for Cramer class 3 of 7.9 μg/kg/d (Yang et al., 2017), whereas the inhalation TTC value for toxic substances is somewhat lower with 2 μg/person/d (Tluczkiewicz et al., 2016). EFSA calculated a value of 1.5 μg/kg/d for the original Munro dataset assigned to Cramer class 3 (EFSA WHO, 2016). This comparison supports the safe application of the Cramer class 3 TTC value to compounds being non-genotoxic carcinogens.

A further extension of Cramer class 3 using data from non-genotoxic carcinogenic herbicides, insecticides, fungicides, and other agrochemicals is available with Heusinkveld et al. (2020). For this list of chemicals, the RepDose DB contains chronic LOEL and/or NOEL values from chronic studies for 95 chemicals. Following the same approach as before, these 95 compounds were assigned to Cramer classes (all were class 3), potentially bioaccumulating compounds were excluded, and the respective fifth NOEL percentile was derived. Steroids were not contained in this dataset. The fifth percentiles obtained are 16 μg/kg /d or 5 × 10^−5^ mmol/kg bw/d. These values are similar to the percentiles derived for the non-genotoxic dataset of this project and, thus, further, substantiate the applicability of Cramer class 3 and the associated TTC value for non-genotoxic carcinogens.

### Robustness of the Threshold of Toxicological Concern Values

The exclusion of substances exhibiting non-human relevant mechanisms, as we did in the data set, is generally accepted (Boobis et al., 2017), although it has been questioned before (EFSA WHO, 2016).

The exclusion of several structural groups from the application of the TTC is generally agreed (Boobis et al., 2017), although reasons for exclusion differ: some structural groups cannot be assessed by the TTC concept because they fall out of the applicability domain. This means that compounds with similar structural properties are not contained in the TTC datasets, e.g., metals, proteins, inorganic salts, polymers, etc. Other compounds, such as dibenzodioxin or-diphenyl-derivatives, are known for their excess toxicity and potential to bioaccumulate. For these compound classes, category-specific TTC values, such as that for organophosphates, do not exist at this time, as the value is likely to be too low to be practically applicable. Nevertheless, some substances from these excluded classes are part of the original Munro dataset, and their NOELs contribute to the respective TTC values. This is due to the fact that only few substances are concerned, and these are scattered over the full range of NOELs.

In the analysis, the exclusion of the potentially bioaccumulating substances from the non-genotoxic data has a significant influence on the TTC value derived from the NOEL values. When the potentially bioaccumulating substances were excluded, the resulting fifth percentile of the residual chemicals is higher than the highest fifth percentile obtained after 100 iterations of removing 20% of the whole dataset randomly. Furthermore, the high toxicity of these substances has already been widely accepted, as this is the reason for excluding this group from TTC application. It is, however, discussed that bioaccumulating substances are within the scope of the TTC concept (Leeman et al., 2016). The definition of bioaccumulating potential, as well as the dataset, was different in this publication, especially the latter being the most probable reason for the different outcomes. Through the application of the method of random leave out, we nicely show in an objective way that the excluded substances are distinct, and the very same reasoning holds true for the steroids.

The method of randomly leaving out a certain number of chemicals not only supports the exclusion of substance groups but also indicates the robustness of the fifth percentile. The smaller the dataset and the wider spread the NOEL values, the higher the range after multiple random trials of leaving out 5% of the substances. The similar ranges of about a factor of 2–3 for all ranges of the TTC values increase the confidence of the authors in the TTC values, as this order of magnitude is approximately similar to one dose spacing difference.

## Conclusion

Existing *in silico* or *in vitro* methods to detect non-genotoxic carcinogen mode of actions are insufficiently accurate yet (Benigni et al., 2013; Papamokos and Silins, 2016). Consequently, when applying the current TTC concept, structures cannot reliably be identified as non-genotoxic carcinogens. Nevertheless, it is commonly agreed upon that a threshold for non-genotoxic compounds can be defined (Kroes et al., 2004). An important question regarding the TTC concept is whether or not the TTC values currently in use also apply to such compounds. The datasets that have been used so far to derive the TTC values contained non-genotoxic carcinogens as well as other substances with chronic toxicity. With the current dataset of non-genotoxic substances alone, supplemented by the dataset used for replication, we were able to show that the application of Cramer class 3 to unidentified non-genotoxic carcinogens is appropriate. Also, the distribution of Cramer class 1 values for non-genotoxic carcinogens falls within the distribution width of the original Munro Cramer class 1 data. Within this process, we confirmed that substances belonging to the cohort of concern should be excluded from the datasets used to derive TTC values, as they heavily influence the fifth percentile by their high toxicity. The robustness of the TTC values in this study was supported by a random leave out analysis of 5% of the dataset compounds.

## Data Availability Statement

The original contributions presented in the study are included in the article/Supplementary Material, further inquiries can be directed to the corresponding author/s.

## Author Contributions

MB and SE contributed to conception and design of the study. FA supported the BMDL calculations. RK supported the statistical analysis. MB wrote the first draft of the manuscript. MC, JR, and CY provided the data set of the LRI B18 project. All authors contributed to manuscript revision, read, and approved the submitted version.

## Conflict of Interest

JR and CY were employed by the company Altamira and Moleculare Networks. The remaining authors declare that the research was conducted in the absence of any commercial or financial relationships that could be construed as a potential conflict of interest.

## Footnotes

^1^Pubchem (2021). https://pubchem.ncbi.nlm.nih.gov/.

^2^Pubmed (2021). https://pubmed.ncbi.nlm.nih.gov/.
