# Supplementary material for: Threshold of Toxicological Concern—An Update for Non-Genotoxic Carcinogens
Source: Front Toxicol. 2021 Jun 24;3:688321. doi: 10.3389/ftox.2021.688321 (PMC8915827; doi:10.3389/ftox.2021.688321)
Supplement: Supplementary file 1 [file Table_1.docx]

Table 1: Non-genotoxic carcinogens with NOEL (mmol/kg bw/d), classification as bioaccumulating or steroid like substance and respective study reference.

| **cas** | **chemical_name** | **NOEL, mmol** | **Bioacc.** | **Steroids** | **Authors** | **Year** | **Journal publication** |
| --- | --- | --- | --- | --- | --- | --- | --- |
| 50282 | 17-beta-Estradiol | 8,74E-07 |  | x | DL Greenman et al. | 1983 | Non-neoplastic changes induced in female C3H mice by chronic exposure to diethylstilbestrol or 17-beta-estradiol. Journal of Toxicology and Environmental Health |
| 50293 | Dichlordiphenyltrichlorethan | 1,34E-03 | x |  | Kashyap, S.K. | 1977 | Int. J. Cancer |
| 50555 | Reserpine | 4,56E-05 |  |  | US Department of Health and Human Services | 1982 | NTP TR 193 |
| 51036 | Piperonyl butoxide | 1,64E-03 |  |  | Sarles, M.P. & Vandegrift, W.B. | 1952 | Am. J. Trop. Med. Hyg. |
| 51525 | Propylthiouracil | 2,80E-03 |  |  | IARC, Monographs 79, 1987: King et al. | 1963 | unpubl. Report |
| 56235 | Carbon tetrachloride | 3,59E-03 |  |  | Nagano, K et al. | 1998 | Chiyotani, K et al. (Eds) Advances in the Prevention of Occ. Resp. Diseases, Amsterdam, Elsevier |
| 56406 | Glycine | 3,27E-01 |  |  | Kitahiro, Y. | 1994 | J Toxicol Pathol |
| 57669 | Probenecid | 1,67E-02 |  |  | US Department of Health and Human Services | 1991 | NTP TR 395 |
| 57681 | Sulfamethazine | 1,28E-01 |  |  | N. A. Littlefield | 1989 | Fd Chem. Toxic. |
| 57749 | Chlordane | 6,78E-05 | x |  | JMPR, Chlordane, 1986: Ihui S. et al. | 1983 | unpubl. rep. by Research Institute for Animal Science in Biochemistry and Toxicology |
| 60355 | Acetamide | 1,48E+00 |  |  | Fleischman, R.W. | 1980 | Journal of environmental pathology and toxicology |
| 60571 | Dieldrin | 4,38E-06 | x |  | Fitzhugh, O. G., A. A. Nelson, et al. | 1964 | Chronic oral toxicity of aldrin and dieldrin in rats and dogs. (Chronic Dieldrin in Rats) Food and Cosmetics Toxicology |
| 61825 | Amitrol | 3,30E-03 |  |  | Jukes T.H. and Shaffer C.B. | 1960 | Science |
| 62533 | Aniline | 8,95E-03 |  |  | BUA 171, Aniline, 1996: CIIT | 1982 | unpubl. rep.: Hazleton Laboratories |
| 63058 | 4-Androstene-3,17-dione | 3,33E-04 |  | x | US Department of Health and Human Services | 2010 | NTP TR 560 |
| 67663 | Chloroform | 5,50E-02 |  |  | NTP (US National Toxicology Program) | 1976 | Technical Report Series |
| 67721 | Hexachloroethane | 3,52E-03 |  |  | NTP (US National Toxicology Program) | 1989 | Technical Report Series |
| 71432 | Benzene | 1,52E-02 |  |  | NTP (US National Toxicology Program) | 1986 | Technical Report Series |
| 72559 | p,p-DDE | 5,82E-03 | (x DDT Metabolit) |  | Tomatis | 1974 | Journal of the National Cancer Institute |
| 75127 | Formamide | 2,11E-02 |  |  | NTP (US National Toxicology Program) | 2008 | Technical Report Series |
| 75650 | tert-Butyl alcohol | 9,56E-02 |  |  | NTP (US National Toxicology Program) | 1995 | Technical Report Series |
| 76017 | Pentachloroethane | 3,08E-02 |  |  | NTP (US National Toxicology Program) | 1983 | Technical Report Series |
| 76039 | Trichloroacetic acid | 1,93E-02 |  |  | Pereira, M.A. | 1996 | TOXICOLOGY AND APPLIED PHARMACOLOGY |
| 76448 | Heptachlor | 1,98E-04 | x |  | S. S. Epstein | 1976 | Science of the Total Environment |
| 77098 | Phenolphthalein | 6,98E-02 |  |  | NTP (US National Toxicology Program) | 1996 | Technical Report Series |
| 78422 | Tris(2-ethylhexyl)phosphate | 5,48E-02 |  |  | NTP (US National Toxicology Program) | 1984 | NTP Toxicology and Carcinogenesis Studies of Tris(2-ethylhexyl) phosphate (CAS No. 78-42-2) In F344/N Rats and B6C3F1 Mice (Gavage Studies). National Toxicology Program technical report series 274: 1-178. (Chronic Mouse) |
| 78591 | Isophorone | 8,61E-02 |  |  | NTP (US National Toxicology Program) | 1986 | Technical Report Series |
| 79005 | 1,1,2-Trichloroethane | 2,87E-02 |  |  |  | 1978 | NTP_1978_Bioassay of 1,1,2-Trichloroethane for Possible Carcinogenicity_rat_TR-74 |
| 79016 | Trichloroethylene | 9,54E-02 |  |  | Maltoni, et al. | 1988 | Ann N Y Acad Sci |
| 79345 | 1,1,2,2-Tetrachloroethane | 2,13E-02 |  |  | NTP (US National Toxicology Program) | 1978 | Technical Report Series |
| 80057 | Bisphenol A | 3,24E-02 |  |  | NTP (US National Toxicology Program) | 1982 | Technical Report Series |
| 80079 | p,p-Dichlorodiphenyl sulfone | 5,81E-04 |  |  | NTP (US National Toxicology Program) | 2001, 2006 | Technical Report Series |
| 80080 | Dapsone | 1,34E-02 |  |  | US Department of Health, Education, and Welfare | 1977 | NTP TR 020 |
| 81152 | Musk xylene | 1,87E-02 |  |  | Maekawa, A et al. | 1990 | Food Chem Toxicol |
| 85687 | Butyl benzyl phthalate | 5,34E-02 |  |  | NTP (US National Toxicology Program) | 1997 | Technical Report Series |
| 87296 | Cinnamyl anthranilate | 3,29E-01 |  |  | NTP (US National Toxicology Program) | 1980 | Technical Report Series |
| 87865 | Pentachlorophenol | 3,13E-03 | (x) |  | NTP (US National Toxicology Program) | 1989 | Technical Report Series |
| 90120 | 1-Methylnaphthalene | 2,40E-02 |  |  | Murata, Y et al. | 1993, 1997 | Fundam Appl Toxicol |
| 94586 | Dihydrosafrole | 3,38E-02 |  |  | Hagan et al. | 1965 | Toxicol. Appl. Pharmacol. |
| 95794 | 5-Chloro-o-toluidine | 7,36E-02 |  |  | NTP (US National Toxicology Program) | 1978 | Technical Report Series |
| 98851 | alpha-Methylbenzyl alcohol | 2,59E-01 |  |  | NTP (US National Toxicology Program) | 1990 | Technical Report Series |
| 99990 | p-Nitrotoluene | 5,07E-02 |  |  | NTP (US National Toxicology Program) | 2002 | Technical Report Series |
| 103231 | Di(2-ethylhexyl) adipate | 2,40E-01 |  |  | NTP (US National Toxicology Program) | 1982 | Technical Report Series |
| 104767 | 2-Ethylhexanol | 1,83E-02 |  |  | Astill et al. | 1996 | Oncogenicity testing of 2-ethylhexanol in Fischer 344 rats and B6C3F1 mice. Fundamental and Applied Toxciology |
| 105555 | N,N'-Diethylthiourea | 1,05E-02 |  |  | NTP | 1979 |  |
| 106467 | p-Dichlorobenzene | 1,43E-02 |  |  | EU RAR, 1,4-Dichlorobenzene, 2004: JISHA | 1995 | Toxicology & carcinogenesis studies |
| 108054 | Vinyl acetate | 4,08E-02 |  |  | IRIS, Vinyl acetate, 1990: Owen, P.E. et al | 1988 | unpubl. Report |
| 108781 | Melamine | 9,91E-02 |  |  | NTP (US National Toxicology Program) | 1983 | Technical Report Series |
| 110861 | Pyridine | 7,37E-03 |  |  | NTP (US National Toxicology Program) | 2000 | Technical Report Series |
| 115286 | 1,4,5,6,7,7-hexachlorobicyclo[2.2.1]hept-5-ene-2,3 | 5,79E-03 |  |  |  | 1987 | NTP Toxicology and Carcinogenesis Studies of Chlorendic Acid (CAS No. 115-28-6) in F344/N Rats and B6C3F1 Mice (Feed Studies) (Chronic Rat). Natl Toxicol Program Tech Rep Ser |
| 115968 | Tris(2-chloroethyl) phosphate | 2,57E-02 |  |  |  | 1991 | NTP Toxicology and Carcinogenesis Studies of Tris(2-chloroethyl) Phosphate (CAS No. 115-96-8) in F344/N Rats and B6C3F1 Mice (Gavage Studies) (Chronic rat). Natl Toxicol Program Tech Rep Ser |
| 117817 | di-sec-octyl Phthalate | 1,90E-03 |  |  | David, R. M. | 2000 | Toxicol Sci |
| 118741 | Hexachlorobenzene | 1,00E-03 | x |  | Cabral, et al. | 1979 | Int J Cancer |
| 119619 | Benzophenone | 6,86E-03 |  |  | NTP (US National Toxcicology Program) | 2007 | Technical Report Series |
| 119846 | 3,4-Dihydrocoumarin | 6,43E-02 |  |  | NTP | 1993 | NTP TR 423 |
| 120321 | o-Benzyl-p-Chlorophenol | 1,14E-02 |  |  |  | 1994 | Biotechnical Services, Inc. prepared technical report. Toxicology and Carcinogenesis Studies of o- Benzyl-p-chlorophenol in F344/N Rats and B6C3F1 Mice (Gavage Studies):Study # NTP TR 424: Battelle Columbus Laboratories Jan. 1994 |
| 120627 | Piperonyl sulfoxide | 7,99E-03 |  |  | U.S. DEPARTMENT OF HEALTH, EDUCATION, AND WELFARE | 1979 | NTP TR 124 |
| 120809 | Catechol | 2,50E-02 |  |  | A. HAGIWARA | 2001 | Toxicologic Pathology |
| 120821 | 1,2,4-Trichlorobenzene | 6,82E-03 |  |  | MAK, 1,2,4-Trichlorobenzene, 2000: Standard Chlorine of Delaware | 1994 | unpubl. Report, project No HWA 2603-102 |
| 121799 | Propyl gallate | 1,57E-01 |  |  | NTP (US National Toxicology Program) | 1982 | Technical Report Series |
| 123353 | Myrcene | 8,74E-02 |  |  |  | 2010 | TOXICOLOGY AND CARCINOGENESIS STUDIES OF beta-MYRCENE (CAS NO. 123-35-3) IN F344/N RATS AND B6C3F1 MICE (GAVAGE STUDIES). NTP TR 557, NIH Publication No. 11-5898. (Mouse Chronic) |
| 123911 | 1,4-Dioxane | 1,51E-02 |  |  | EU RAR 21, 2002: Yamazaki K | 1994 | unpubl. Report |
| 126738 | Tributyl phosphate | 1,46E-02 |  |  | Auletta, CS et al. | 1998 | Toxicology |
| 127184 | Tetrachloroethene | 7,29E-02 |  |  | NTP (US National Toxicology Program) | 1986 | Technical Report Series |
| 127479 | Retinyl acetate | 3,74E-02 |  |  | Kurokawa et al. | 1985 | JNCI: Journal of the National Cancer Institute |
| 128370 | 2,6-Di-tert-butyl-p-cresol | 6,30E-02 |  |  | Hirose, M et al. | 1981 | Food Chemical Toxicology |
| 128665 | C.I. Vat Yellow 4 | 2,78E-01 |  |  | US Department of Health, Education and Welfare | 1979 | NTP TR 134 |
| 135239 | Methapyrilene hydrochloride | 7,13E-03 |  |  | Lijinsky, W. | 1984 | Fd Chem. Toxic. |
| 136403 | Phenazopyridine hydrochloride | 1,78E-02 |  |  | NTP | 1978 |  |
| 139059 | Sodium cyclamate | 1,48E-01 |  |  | Rudali | 1969 | Academie des Sciences, Memoires et Communications des Membres et des Correspondants de l&#039;Academie |
| 139139 | Nitrilotriacetic acid | 2,18E-01 |  |  | US Department of Health, Education and Welfare | 1977 | NTP TR 6 |
| 140114 | Benzyl acetate | 1,63E-02 |  |  | NTP (US National Toxicology Program) | 1993 | Technical Report Series |
| 143500 | Kepone | 1,13E-05 | x |  | Chu et. al. | 1981 | Toxicology and Applied Pharmacology |
| 148798 | Thiabendazole | 1,64E-03 |  |  | Bagdon, W.J.; Bokelman, D.L.; Zwickey, R.E.; Et Al. | 1980 | Thiaben- Dazole: Lifetime Carcinogenic Study In Mice: Tt # 77-014-0. (Unpublished Study Received Apr 1, 1980 Under 618-75; Submitted By Merck & Co., Inc., Rahway, N.J.; Cdl:242116-A) |
| 149304 | 2-Mercaptobenzothiazole | 9,37E-02 |  |  | NTP (US National Toxicology Program), Dieter, MP | 1988 | Technical Report Series |
| 150685 | Monuron | 1,57E-02 |  |  |  | 1988 | TOXICOLOGY AND CARCINOGENESIS STUDIES OF MONURON (CAS NO. 150-68-5) IN F344/N RATS AND B6C3Fi MICE (FEED STUDIES). NTP TR 266, NIH Pub No. 88-2522. (Chronic Rat) |
| 298599 | methylphenidate hydrochloride | 1,19E-02 |  |  | NTP | 1995 |  |
| 309002 | (1R,4S,4aS,5S,8R,8RaR)-1,2,3,4,10,10-hexachloro-1,4,4a,5,8,,8a-hexahydro-1,4:5,8-dimethanonaphthalene | 7,61E-06 | x |  | Fitzhugh, OG et al. | 1964 | Food and Chemical Toxicology |
| 319846 | alpha-1,2,3,4,5,6-Hexachlorocyclohexane | 1,91E-02 | x |  | Ito et al. | 1975 | Journal of the National Cancer Institute |
| 389082 | nalidixic acid | 4,79E-02 |  |  | US Department of Health and Human Services | 1989 | NTP TR 368 |
| 396010 | triamterene | 2,92E-03 |  |  | NTP | 1993 | NTP TR 420 |
| 434071 | Oxymetholone | 7,52E-04 |  | x | National Toxicology Program | 1999 | NTP TECHNICAL REPORT Nr 485 |
| 501304 | Kojic acid | 7,82E-01 |  |  | Fujimoto, N. | 1998 | Food and Chemical Toxicology |
| 510156 | Chlorobenzilate | 1,37E-02 |  |  | Innes J.R.M. et al. | 1969 | J. Natl. Cancer Inst. |
| 510156 | Chlorobenzilate | 1,37E-02 |  |  | NTIS | 1968 | Evaluation of Carcinogenic, Teratogenic, and Mutagenic Activities of Selected Pesticides and Industrial Chemicals |
| 562107 | Doxylamine succinate | 3,62E-03 |  |  | Jackson, C.D. | 1993 | JOURNAL OF THE AMERICAN COLLEGE OF TOXICOLOGY |
| 597251 | Dimethyl morpholinophosphoramidate | 6,41E-02 |  |  | NTP | 1986 |  |
| 598550 | n-Methylcarbamate | 1,11E-01 |  |  |  | 1987 | TOXICOLOGY AND CARCINOGENESIS STUDIES OF METHYL CARBAMATE (CAS NO. 598-55-0) IN F344/N RATS AND B6C3F1 MICE (GAVAGE STUDIES). NTP TR 328, NIH Publication No. 88-2584. (Chronic rat) |
| 604751 | Oxazepam | 1,99E-03 |  |  |  | 1993 | TOXICOLOGY AND CARCINOGENESIS STUDIES OF OXAZEPAM (CAS NO. 604-75-1) IN SWISS-WEBSTER AND B6C3Fl MICE (FEED STUDIES). NTP TR 443, NIH Publication No. 93-3359. (Chronic B6C3F1) |
| 628024 | Hexanamide | 5,43E-01 |  |  | Fleischman et al. | 1980 | Carcinogenesis bioassay of acetamide, hexanamide, adipamide, urea and p-tolylurea in mice and rats_628-02-4_4300_hexanamide_rat_PMID7441078 |
| 628944 | Adipamide | 6,94E-01 |  |  | Fleischman et al. | 1980 | Carcinogenesis bioassay of acetamide, hexanamide, adipamide, urea and p-tolylurea in mice and rats_628-94-4_4300_adipamide_rat_PMID7441078 |
| 630206 | 1,1,1,2-Tetrachloroethane | 6,21E-02 |  |  | NTP (US National Toxicology Program) | 1983 | Technical Report Series |
| 634935 | 2,4,6-Trichloroaniline | 2,26E-01 |  |  | Weisburger, E.K. | 1978 | Journal of Environmental Pathology &amp; Toxicology |
| 693981 | 2-Methylimidazole | 2,03E-02 |  |  | U.S. DEPARTMENT OF HEALTH AND HUMAN SERVICES | 2004 | NTP TECHNICAL REPORT Nr 516 |
| 822366 | 4-Methylimidazole | 2,32E-02 |  |  | NTP (US National Toxicology Program) | 2007 | Technical Report Series |
| 872504 | N-Methyl-2-pyrrolidone | 8,31E-03 |  |  | Lee, KP, et al. | 1987 | Fundam Appl Toxicol |
| 961115 | Tetrachlorvinphos | 4,84E-02 |  |  |  | 1978 | BIOASSAY OF TETRACHLORVINPHOS FOR POSSIBLE CARCINOGENICITY (CAS No. 961-11-5). Study No. NCI-CG-TR-33. (Rat portion) |
| 1163195 | Decabromodiphenyl oxide | 1,45E-01 |  |  | NTP (US National Toxicology Program) | 1986 | Technical Report Series |
| 1596845 | Daminozide | 1,27E-02 |  |  | Johnson, D. | 1988 | Alar Technical (Daminozide): Two Year Oncogenicity Study In Mice: Report No. 399-054. Unpublished Study Prepared By International Research And Development Corp. |
| 1634044 | Methyl-tertiary-butyl ether | 2,36E-01 |  |  | Belpoggi F et al. | 1995 | Toxicology and Ind. Health |
| 1746016 | 2,3,7,8-Tetrachlorodibenzo-p-dioxin | 1,81E-11 | x |  | van Miller, J. P. | 1977 | Chemosphere |
| 1897456 | Chorothalonil | 1,57E-04 |  |  | Ford, W.; Laveglia, J.; Killeen, J.; Et Al. | 1983 | A Two-Year Toxicity And Tumorigenicity Study Of Ds-3701 In Rats: Document Num- Ber 100-5Tx-80-0016-011. Final Rept. (Unpublished Study Re- Ceived Jan 10, 1984 Under 50534-8; Submitted By Sds Biotech Cor |
| 1912249 | Atrazine | 1,35E-03 |  |  | Hardisty, J. | 1987 | Supplement to Two-year Chronic Feeding/Oncoge- nicity Study in Rats Administered Atrazine: 410-1102. Unpub- lished study prepared by Experimental Pathology Laboratories, Inc. |
| 2385855 | Mirex | 1,07E-06 | x |  | US Department of Health and Human Services | 1990 | NTP TR 313 |
| 2432997 | 11-Aminoundecanoic acid | 2,07E-01 |  |  | NTP (US National Toxicology Program) | 2004 | Technical Report Series |
| 2489772 | 1,1,3-Trimethyl-2-thiourea | 1,18E-02 |  |  | NTP | 1979 |  |
| 2835394 | Allyl isovalerate | 1,04E-02 |  |  | NTP (US National Toxicology Program) | 1983 | Technical Report Series |
| 3347226 | Dithianon | 2,25E-03 |  |  | JMPR Monography 843, Dithianon, 1992: Brown, D | 1991 | Unpublished report No. 6165-460/14 dated August 1991 from Hazleton UK, Harrogate, England cited from JMPR (1992) |
| 3546109 | Phenesterin | 6,46E-04 |  |  | US Department of Health, Education and Welfare | 1978 | NTP TR 60 |
| 3564098 | Ponceau 3R | 3,37E-02 |  |  | Grice, H.C. | 1961 | TOXICOLOGY AND APPLIED PHARMACOLOGY |
| 4180238 | trans-Anethole | 9,37E-02 |  |  | Truhaut, R et al. | 1989 | Food Chem Toxicol |
| 5392405 | Citral | 1,88E-02 |  |  | NTP (US National Toxicology Program) | 2001 | Technical Report Series |
| 5989275 | d-Limonene | 4,59E-02 |  |  | NTP (US National Toxicology Program) | 1990 | Technical Report Series |
| 8015303 | Enovid | 1,37E-05 |  | x | Poel | 1966 | Science |
| 11096825 | Aroclor 1260 | 3,88E-04 | x |  | Mayes, B. A. et al. | 1998 | Toxological Sciences |
| 11097691 | Aroclor 1254 | 4,26E-04 | x |  | U.S. DEPARTMENT OF HEALTH, EDUCATION, AND WELFARE | 1978 | NTP TR 38 |
| 12674112 | Aroclor 1016 | 1,09E-03 | x |  | Mayes, B. A. et al. | 1998 | Toxological Sciences |
| 13073353 | Ethionine | 2,01E-03 |  |  | Ogiso, T. | 1985 | Toxicologic Pathology |
| 17924924 | Zeralenone | 3,27E-04 |  |  |  | 1982 | CARCINOGENESIS BIOASSAY OF ZEARALENONE (CAS NO. 17924-92-4) IN F344/N RATS AND B6C3F1 MICE (FEED STUDY). NTP-81-54, NIH Publication No. 83-1791. (Chronic rat) |
| 18662538 | NTA trisodium salt dihydrate | 3,57E-02 |  |  | Goyer, R.A. | 1981 | Journal of the National Cancer Institute |
| 25013165 | Butylated hydroxyanisole | 3,83E-02 |  |  | Ito, N et al. | 1986 | Food Chem Toxicol |
| 31508006 | 1,2,4-trichloro-5-(3,4-dichlorophenyl)benzene | 2,55E-05 | x |  | NTP | 2010 |  |
| 37319178 | Elmiron | 3,47E-03 |  |  | U.S. DEPARTMENT OF HEALTH AND HUMAN SERVICES | 2004 | NTP TR 512 |
| 39148248 | Fosetyl-aluminium | 3,53E-01 |  |  | Quest, J. A. | 1991 | Regulatory Toxicology and Pharmacology |
| 39801144 | Photomirex | 2,20E-06 | x |  | I. Chu et al. | 1981 | Toxicology and Applied Pharmacology |
| 53469219 | Aroclor 1242 | 1,07E-03 | x |  | Mayes, B. A. et al. | 1998 | Toxological Sciences |
| 57465288 | 1,2,3-trichloro-5-(3,4-dichlorophenyl)benzene | 7,66E-09 | x |  | NTP | 2006 |  |
| 63449398 | Chlorinatedparaffins | 1,10E-02 |  |  | US Department of Health and Human Services | 1986 | NTP TR 308 |
| 67774327 | Polybrominated biphenyls | 3,85E-04 | x |  | U.S. DEPARTMENT OF HEALTH AND HUMAN SERVICES | 1993 | NTP 398 |
| 68515480 | Di-isononyl phthalate (DINP1) | 3,96E-03 |  |  | Lington AW et al. | 1997 | Fundamental and Applied Toxicology |
| 76231760 | (-)-alpha,beta-Thujone | 7,82E-03 |  |  | US Department of Health and Human Services | 2011 | NTP TR 570 |
| 79520777 | 5,5-(1,1-BIPHENYL)-2,5-DYLBIS(OXY)(2,2-DIMETHYLPENTANOIC ACID) | 5,38E-04 |  |  | Walker et al. | 1996 | Toxicol. Pathol. |
| 86386734 | Fluconazole | 4,54E-05 |  |  | Paulus, G. | 1994 | Teratogenesis, Carcinogenesis, and Mutagenesis |
| 116355830 | Fumonisin b1 | 7,70E-06 |  |  | Gelderblom, W.C.A. | 2001 | Toxicology |
| 123312890 | Pymetrozine | 2,63E-04 |  |  | Gerspach, R. | 1995 | Cga-215944 Technical: 18-Month Carcinogenicity Study In Mice: Final Report: Lab Project Number: 901482: Tox.0306A3Cb. Unpublished Study Prepared By Ciba-Geigy Ltd. |
| 210631688 | Topramezone | 9,17E-05 |  |  | Kaspers, U.; Deckardt, K.; Kuttler, K.; Et Al. | 2003 | Bas 670 H--Carcogenicity Study In Wistar Rats Administration In The Diet For 24 Months: Final Report: Lab Project Number: 82S0124/98099: 2003/1006262: 2003/1001455. Unpublished Study Prepared By Basf A |
