# Supplementary material for: Threshold of Toxicological Concern—An Update for Non-Genotoxic Carcinogens
Source: Front Toxicol. 2021 Jun 24;3:688321. doi: 10.3389/ftox.2021.688321 (PMC8915827; doi:10.3389/ftox.2021.688321)
Supplement: Supplementary file 2 [file Table_2.DOCX]

Supplement 2: NOEL, BMDL and ETD10 values (mg/kg bw/d) with indication of exclusion categories (Bioaccumulating in humans, Steroids)

| CAS | Chemical | NOEL, mg/kg bw/d | Bioacc. | Steroids | BMDL, mg/kg bw/d | ETD10, mg/kg bw/d |
| --- | --- | --- | --- | --- | --- | --- |
| 50282 | 17beta-Estradiol | 0,00024 |  | x | 0,03857 |  |
| 2385855 | Mirex | 0,00058 | x |  | 0,00065 | 0,55022 |
| 50555 | Reserpine | 0,00086 |  |  | 0,00300 | 0,12274 |
| 39801144 | Photomirex | 0,00111 | x |  | 0,03000 | 0,12014 |
| 55389 | Fenthion | 0,00143 |  |  | 0,04143 |  |
| 60571 | Dieldrin | 0,00167 | x |  | 0,01043 | 0,04201 |
| 303479 | Ochratoxin a | 0,00175 |  |  | 0,00036 | 0,00379 |
| 309002 | (1R,4S,4aS,5S,8R,8RaR)-1,2,3,4,10,10-hexachloro-1,4,4a,5,8,,8a-hexahydro-1,4:5,8-dimethanonaphthalene | 0,00278 | x |  | 0,01237 |  |
| 143500 | Kepone | 0,00556 | x |  | 0,02000 | 0,17763 |
| 116355830 | fumonisin b1 | 0,00556 |  |  | 0,18571 | 0,30922 |
| 8015303 | Enovid | 0,00833 |  | x | 0,00557 |  |
| 72208 | Endrin | 0,01185 | x |  | 0,00307 | 0,00557 |
| 86386734 | Fluconazole | 0,01389 |  |  | 0,94571 | 2,35884 |
| 115093 | Methylmercuric(II) chloride | 0,01481 |  |  | 0,12286 | 0,16845 |
| 56382 | Parathion | 0,02778 |  |  | 0,02457 | 0,16962 |
| 57749 | chlordane | 0,02778 | x |  | 0,04000 | 0,00915 |
| 96457 | Ethylene thiourea | 0,02778 |  |  | 1,10857 | 0,38126 |
| 56359 | Tributyltin oxide | 0,03333 |  |  | 0,00054 | 0,11888 |
| 87865 | Pentachlorophenol | 0,04084 | (x) |  | 0,27143 | 0,82015 |
| 87865 | Pentachlorophenol | 0,04084 | (x) |  | 0,27143 | 1,19034 |
| 118741 | Hexachlorobenzene | 0,04167 | x |  | 1,71429 | 2,19623 |
| 1897456 | Chorothalonil | 0,04167 |  |  | 23,57143 | 73,27513 |
| 2164172 | Fluometuron | 0,04583 |  |  | 2,00000 |  |
| 75605 | Dimethylarsinic acid | 0,06083 |  |  | 0,15714 | 0,85160 |
| 76448 | Heptachlor | 0,07405 | x |  | 0,06000 | 0,12232 |
| 17924924 | Zeralenone | 0,10417 |  |  | 0,26000 | 0,02577 |
| 11097691 | Aroclor 1254 | 0,13891 | x |  | 0,01571 | 0,81861 |
| 11096825 | Aroclor 1260 | 0,13891 | x |  | 0,04314 | 0,34329 |
| 80079 | p,p-Dichlorodiphenyl sulfone | 0,16670 |  |  | 0,15571 | 5,59644 |
| 79345 | 1,1,2,2-Tetrachloroethane | 0,17914 |  |  | 1,01286 | 1,79201 |
| 54965241 | Tamoxifen citrate | 0,23333 |  |  | 0,11429 | 0,31708 |
| 79520777 | 5,5&#039;-(1,1&#039;-BIPHENYL)-2,5-DYLBIS(OXY)(2,2-DIMETHYLPENTANOIC ACID) | 0,23810 |  |  | 0,13243 | 3,71579 |
| 434071 | Oxymetholone | 0,25000 |  | x | 0,09314 | 6,06666 |
| 61825 | Amitrol | 0,27783 |  |  | 0,05286 | 0,11560 |
| 12674112 | Aroclor 1016 | 0,27783 | x |  | 0,82143 | 2,21004 |
| 1912249 | Atrazine | 0,29167 |  |  | 0,01957 | 6,52050 |
| 148798 | Thiabendazole | 0,33095 |  |  | 12,08571 | 28,30632 |
| 3546109 | Phenesterin | 0,41667 |  |  | 0,02857 | 0,12011 |
| 50293 | Dichlordiphenyltrichlorethan | 0,47619 | x |  | 0,00414 | 1,04366 |
| 51525 | Propylthiouracil | 0,47619 |  |  | 11,57143 | 89,54962 |
| 51036 | Piperonyl butoxide | 0,55566 |  |  | 1,30000 | 22,28809 |
| 604751 | Oxazepam | 0,57143 |  |  | 0,15714 | 4,97437 |
| 110861 | Pyridine | 0,58333 |  |  | 0,15143 | 2,50408 |
| 6459945 | C.I. Acid Red 114 | 0,68757 |  |  | 0,06429 | 0,23777 |
| 117817 | di-sec-octyl Phthalate | 0,74048 |  |  | 0,10286 | 24,99928 |
| 396010 | triamterene | 0,74048 |  |  | 0,15857 |  |
| 872504 | N-Methyl-2-pyrrolidone | 0,82400 |  |  | 30,00000 | 37,64829 |
| 67721 | Hexachloroethane | 0,83333 |  |  | 1,29143 | 2,96466 |
| 120616 | Dimethyl terephthalate | 1,15317 |  |  | 10,12857 | 35,64265 |
| 37319178 | elmiron | 1,16667 |  |  | 7,25714 | 2,59897 |
| 71432 | Benzene | 1,19048 |  |  | 0,22857 | 0,34719 |
| 98319267 | Finasteride | 1,19048 |  | x | 14,00000 | 24,64663 |
| 123911 | 1,4-Dioxane | 1,33333 |  |  | 1,71429 | 12,53961 |
| 105555 | N,N'-Diethylthiourea | 1,38915 |  |  | 0,75714 | 1,84929 |
| 562107 | Doxylamine succinate | 1,40691 |  |  | 1,21429 | 10,56582 |
| 2835394 | Allyl isovalerate | 1,47619 |  |  | 2,71429 | 7,27533 |
| 68515480 | Di-isononyl phthalate (DINP1) | 1,66698 |  |  | 0,85429 | 100,73251 |
| 693981 | 2-Methylimidazole | 1,66698 |  |  | 1,11429 | 46,60745 |
| 72559 | p,p-DDE | 1,85120 | (x DDT Metabolit) | | 0,32857 | 0,56401 |
| 1596845 | Daminozide | 2,04095 |  |  | 17,57143 | 77,30283 |
| 57410 | 5,5-Diphenylhydantoin | 2,08333 |  |  | 0,76571 | 8,56816 |
| 106467 | p-Dichlorobenzene | 2,10595 |  |  | 1,71429 | 12,90013 |
| 135239 | Methapyrilene hydrochloride | 2,12500 |  |  | 0,09286 | 2,55230 |
| 115286 | 1,4,5,6,7,7-hexachlorobicyclo[2.2.1]hept-5-ene-2,3 | 2,25000 |  |  | 0,28571 | 8,00279 |
| 140114 | Benzyl acetate | 2,44358 |  |  | 3,42857 | 15,05395 |
| 120321 | o-Benzyl-p-Chlorophenol | 2,50000 |  |  | 9,94286 | 37,01828 |
| 67663 | Chloroform | 2,53276 |  |  | 4,28571 | 3,64263 |
| 81152 | Musk xylene | 2,57143 |  |  | 1,57143 | 4,07173 |
| 120627 | Piperonyl sulfoxide | 2,59168 |  |  | 0,80000 |  |
| 120809 | Catechol | 2,75000 |  |  | 6,14286 | 2,28772 |
| 298599 | methylphenidate hydrochloride | 2,77830 |  |  | 0,27143 | 6,40107 |
| 5392405 | Citral | 2,85714 |  |  | 5,85714 | 8,06748 |
| 150685 | Monuron | 3,12500 |  |  | 1,22286 | 5,73977 |
| 76039 | Trichloroacetic acid | 3,14964 |  |  | 13,85714 | 34,34340 |
| 80080 | Dapsone | 3,33395 |  |  | 0,34286 | 2,36137 |
| 90120 | 1-Methylnaphthalene | 3,40952 |  |  | 1,85714 | 2,59499 |
| 108054 | Vinyl acetate | 3,51402 |  |  | 2,57143 | 15,87399 |
| 115968 | Tris(2-chloroethyl) phosphate | 3,66667 |  |  | 2,00000 | 6,50081 |
| 79005 | 1,1,2-Trichloroethane | 3,83333 |  |  | 4,12857 | 0,23286 |
| 510156 | Chlorobenzilate | 4,46509 |  |  | 2,00000 | 21,55212 |
| 57669 | probenecid | 4,76190 |  |  | 12,08571 | 9,37216 |
| 28407376 | c.i. direct blue 218 | 5,55659 |  |  | 1,08429 | 1,77130 |
| 63449398 | Chlorinatedparaffins | 5,95238 |  |  | 3,28571 |  |
| 76017 | Pentachloroethane | 6,25000 |  |  | 1,34286 | 16,47463 |
| 5989275 | d-Limonene | 6,25000 |  |  | 1,57143 | 13,82252 |
| 99990 | p-Nitrotoluene | 6,94574 |  |  | 4,85714 |  |
| 25013165 | Butylated hydroxyanisole | 6,94574 |  |  | 54,28571 | 47,88179 |
| 599791 | Salicylazosulfapyridine | 7,00000 |  |  | 4,14286 | 55,23211 |
| 75650 | tert-Butyl alcohol | 7,08333 |  |  | 365,71429 | 234,18546 |
| 119846 | 3,4-Dihydrocoumarin | 9,52381 |  |  | 2,28571 | 80,72981 |
| 18662538 | NTA trisodium salt dihydrate | 9,82236 |  |  | 18,57143 | 123,52970 |
| 79016 | Trichloroethylene | 10,33333 |  |  | 0,41429 | 101,25941 |
| 79016 | Trichloroethylene | 10,33333 |  |  | 0,41429 | 179,18808 |
| 95794 | 5-Chloro-o-toluidine | 10,41667 |  |  | 2,14286 | 15,75011 |
| 630206 | 1,1,1,2-Tetrachloroethane | 10,41667 |  |  | 4,57143 | 1,71129 |
| 81492 | 1-Amino-2,4-dibromoanthraquinone | 11,11318 |  |  | 0,48571 | 1,31053 |
| 389082 | nalidixic acid | 11,11318 |  |  | 1,08571 | 10,06920 |
| 78591 | Isophorone | 11,90476 |  |  | 5,71429 | 12,74627 |
| 127184 | Tetrachloroethene | 12,08333 |  |  | 0,52857 |  |
| 127479 | retinyl acetate | 12,27795 |  |  | 1,38286 | 5,11054 |
| 597251 | dimethyl morpholinophosphoramidate | 12,50000 |  |  | 2,42857 |  |
| 108781 | Melamine | 12,50233 |  |  | 24,28571 | 44,04376 |
| 59820438 | hc yellow 4 | 13,89148 |  |  | 2,80000 |  |
| 128370 | 2,6-Di-tert-butyl-p-cresol | 13,89148 |  |  | 13,10000 |  |
| 4180238 | trans-Anethole | 13,89148 |  |  | 39,42857 | 123,23581 |
| 1582098 | Trifluralin | 14,08333 |  |  | 4,85714 | 7,16157 |
| 85687 | Butyl benzyl phthalate | 15,00000 |  |  | 36,57143 | 69,21410 |
| 149304 | 2-Mercaptobenzothiazole | 15,66667 |  |  | 1,85714 |  |
| 3564098 | Ponceau 3R | 16,66977 |  |  | 4,14286 | 134,77034 |
| 961115 | Tetrachlorvinphos | 17,70833 |  |  | 124,00000 | 32,93764 |
| 1634044 | Methyl-tertiary-butyl ether | 20,83333 |  |  | 1,71429 | 25,73149 |
| 77098 | Phenolphthalein | 22,21436 |  |  | 3,14286 | 75,22862 |
| 78422 | Tris(2-ethylhexyl)phosphate | 23,80952 |  |  | 11,54286 | 37,04535 |
| 56406 | Glycine | 24,55589 |  |  | 28,57143 |  |
| 33857260 | 2,7-Dichlorodibenzo-P-dioxin | 27,78296 |  |  | 9,71429 |  |
| 139059 | Sodium cyclamate | 29,76190 |  |  | 36,85714 | 17,58646 |
| 98851 | alpha-Methylbenzyl alcohol | 31,66667 |  |  | 4,85714 | 27,80449 |
| 121799 | Propyl gallate | 33,33955 |  |  | 8,91429 | 116,94908 |
| 57681 | Sulfamethazine | 35,54297 |  |  | 32,42857 | 46,54129 |
| 2432997 | 11-Aminoundecanoic acid | 41,67443 |  |  | 7,10000 | 47,39869 |
| 139139 | Nitrilotriacetic acid | 41,67443 |  |  | 22,71429 | 26,74612 |
| 634935 | 2,4,6-Trichloroaniline | 44,42872 |  |  | 9,55714 | 27,30298 |
| 7177482 | Ampicillin trihydrate | 62,50000 |  |  | 11,85714 |  |
| 628024 | Hexanamide | 62,50000 |  |  | 27,57143 | 146,80557 |
| 87296 | Cinnamyl anthranilate | 83,34887 |  |  | 14,28571 | 11,04108 |
| 60355 | Acetamide | 87,37648 |  |  | 40,00000 | 288,11611 |
| 103231 | Di(2-ethylhexyl) adipate | 88,85744 |  |  | 21,42857 | 59,57686 |
| 128665 | C.I. Vat Yellow 4 | 92,55983 |  |  | 41,42857 | 105,59013 |
| 501304 | Kojic acid | 111,07180 |  |  | 17,14286 | 17,89970 |
| 39148248 | Fosetyl-aluminium | 125,00000 |  |  | 94,28571 | 214,56561 |
| 1212299 | N,N'-Dicyclohexylthiourea | 138,91478 |  |  | 34,28571 | 501,92739 |
| 1163195 | Decabromodiphenyl oxide | 138,91478 |  |  | 42,57143 | 165,65238 |
| 69658 | D-Mannitol | 138,91478 |  |  | 628,57143 |  |
